# Terbium Ion Adsorption from Aqueous Solution by Using Magnetic γ-Fe_2_O_3_-NH_4_OH@SiO_2_ Nanoparticles Functionalized with Amino Groups

**DOI:** 10.3390/ma12081294

**Published:** 2019-04-19

**Authors:** Tina Kegl, Aljoša Košak, Aleksandra Lobnik, Irena Ban

**Affiliations:** 1 Faculty of Chemistry and Chemical Engineering, University of Maribor, Smetanova 17, SI-2000 Maribor, Slovenia; irena.ban@um.si; 2 Institute for Environmental Protection and Sensors, Beloruska 7, SI-2000 Maribor, Slovenia; aljosa.kosak@um.si; 3 Faculty of Mechanical Engineering, University of Maribor, Smetanova 17, SI-2000 Maribor, Slovenia

**Keywords:** rare earth elements, core@shell nanoparticles, adsorption, magnetic separation, kinetic models

## Abstract

New magnetic stabilized and functionalized core@shell nanoparticles (NPs) were synthesized in a simple way and characterized in order to adsorb Tb^3+^ from aqueous solution with a very low Tb^3+^ concentration. For the fluorescence determination of adsorption efficiency and capacity, tiron monohydrate as a ligand was used. The obtained results confirm the potential of the synthesized magnetic γ-Fe_2_O_3_-NH_4_OH@SiO_2_ NPs, functionalized with (3-Aminopropyl) trimethoxysilane (APTMS), to be used for adsorption of Tb^3+^ from aqueous solution, with the possibility of its removal from aqueous solution via an external magnet. The endothermic and spontaneous adsorption follows a pseudo-second-order kinetic model, and the adsorption equilibrium data fit the Temkin isotherm well. The maximum adsorption efficiency from aqueous solution with a 2 × 10^−6^ M concentration of Tb^3+^ is over 90% at pH 7.

## 1. Introduction

Rare earth elements (REEs) are used as components in the energy and transport sectors, environmental protection, digital technology, and medical applications in order to reduce energy consumption, improve mobility and reduce weight, reduce emissions, improve lifestyle and communication, improve medical therapy, and improve life [1,2,3,4]. Due to their low natural abundance and high production demands, Nd, Y, Eu, Tb, and Dy are considered to be the most economically critical REEs [5]. The balance between the natural abundances of the REEs in ores and the economic market demand is a major balance problem for producers of these elements [6,7]. The main source of REEs is China, which has abundant REEs reserves and advanced processing technologies [7,8,9]. In order to solve the balance problem as well as to reduce the dependence on China, the recycling market for REEs has great potential due to the increasing consumption of REE-containing products. As REEs are important for many electronic products, many nations have started looking for alternative supplies of these elements. One potential source of REEs-containing waste is scrapped electronics. In E.U. countries, electronic waste could in theory cover a significant part of the demand for REEs. Furthermore, recycling of REEs should be an important part of a circular economy, especially considering the large resource demands and negative environmental impact of current REEs production processes.

The REEs recycling methods include pyro-metallurgical, electrochemical, and hydrometallurgical approaches [10,11]. Hydrometallurgical methods, such as precipitation, solvent extraction, and adsorption, play a major part in any process developed to recover REEs from a solution. Precipitation and solvent extraction are well-established techniques that are particularly useful for solutions containing high concentrations of the target REE^3+^, and adsorption can be used to recover REE^3+^ even from low-concentration sources [12,13,14,15]. Among the available methods, adsorption has gained wider attention because of its simplicity, high efficiency, and low cost [12,16]. In general, adsorption can either be physical or chemical. While by physical adsorption the adsorbate adheres to the surface only through weak intermolecular Van der Waals interactions in the low-temperature environment and under appropriate pH conditions, by chemical adsorption the adsorbate molecules are held to the solid by new chemical bonds (covalent, ionic) due to strong interactions between adsorbent and adsorbate [17]. The amount of heat produced, when one mole of the adsorbate is adsorbed on the adsorbent’s surface, is the enthalpy of adsorption. Adsorption is usually described through kinetic models and adsorption isotherms.

In recent years, the use of nanoparticles (NPs) as adsorbents has become very popular despite of potential health and safety hazards of some NPs [18,19,20,21]. Since adsorption is an effective, economical, and ecofriendly technique, the adsorption of REE^3+^ from aqueous solution by NPs is one of the best technologies for REE^3+^ removal from aqueous solution. Adsorption is a mass-transfer process, where REE^3+^ are transferred from the solution to the NP surface and become bonded by physical and/or chemical interactions depending on the type of NPs. Adsorption studies are conducted by mixing NPs as an adsorbent with REE^3+^ solution at the desired concentration. The mixtures are stirred with appropriate speed at some fixed temperature for a suitable time and the results are adsorbed REE^3+^ on the NPs (REE^3+^/NPs). Then, the non-magnetic NPs with adsorbed REE^3+^ can be separated by using column extraction, centrifugation, or membrane filtration [22,23,24]. Meanwhile, the magnetic NPs with adsorbed REE^3+^ can simply be removed by using an external magnet [25,26,27]. After removal of REE^3+^/NPs from aqueous solution, the concentration of REE^3+^ in the residual solution is measured in order to determine the adsorption capacity and efficiency [24,28].

To date, for adsorption of Tb^3+^, which is often used in phosphor production for its high number of green emissions that, combined with other lanthanides, produce white-emitting materials for light-emitting diodes (LEDs) [29,30], various non-magnetic and magnetic NPs have been synthesized successfully. By using poly(acrylic acid)-silica hydrogel nanofibers (PAA/SiO_2_ HNFs), the maximum adsorption capacity was 250 mg/g at pH 6 [31]. Double (silica and poly(allylamine) hydrochloride)-coated magnetic NPs, functionalized with diethylenetriaminepentaacetic acid (FeO@SiO_2_@PA/SiO_2_(DTPA) NPs), offer an adsorption capacity of 1.4 mg/g [20]. Yesiller and coauthors investigated the percentage uptake of terbium ions on magnetic NPs, and they showed a 100% uptake at pH 3 on zero valent iron (Fe^0^) NPs and a 95.98% uptake at pH 3 on alumina-supported zero valent iron (Fe^0^/Al) NPs [32]. Wang studied the adsorption capacity of various REEs on montmorillonite-supported zero-valent iron magnetic NPs (n-ZVI-M NPs). The maximum adsorption capacities were found to be 315.23 mg/g for Tb^3+^, which is the highest Tb^3+^ adsorption capacity among magnetic NPs [33]. Experimental data on terbium ions adsorption with all mentioned NPs, except for adsorption on n-ZVI-M NPs, which is best described by the Freundlich adsorption isotherm, were very well-fitted to the Langmuir isotherm model and the adsorption kinetics followed a pseudo-second order model [20,31,33].

This paper deals with the adsorption of Tb^3+^ from aqueous solutions onto very simple magnetic γ-Fe_2_O_3_-NH_4_OH@SiO_2_(APTMS) core@shell NPs, which enable simple removal of a low concentration of Tb^3+^ from aqueous solution with high adsorption efficiency. The dependence of adsorption parameters, such as contact time, mass of NPs, temperature, and initial concentration of Tb^3+^, on the adsorption efficiency was studied. The adsorption process took place in a time range of up to 1440 min, and the mass of NPs was varied from 5 mg to 40 mg; the volume of the solution was 20 mL. Meanwhile, initial concentration ranged from 5 × 10^−7^ to 4 × 10^−6^ M. Experiments were carried out at three different temperatures: 25 °C, 50 °C, and 75 °C. Furthermore, the adsorption kinetics and adsorption isotherms were analyzed by using nonlinear methods, and the thermodynamic parameters were derived from the obtained experimental results.

## 2. Results and Discussion

### 2.1. γ-Fe_2_O_3_-NH_4_OH@SiO_2_(APTMS) NPs

**Figure 1:**
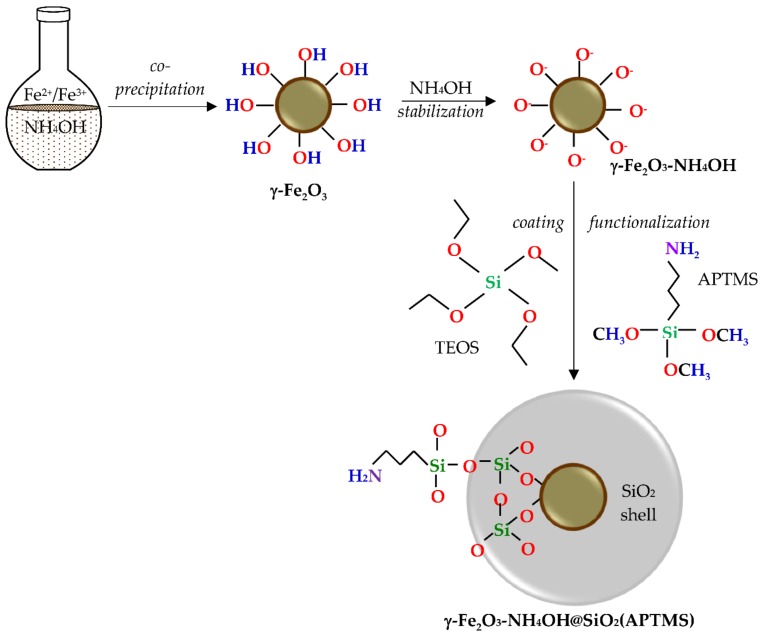
Magnetic γ-Fe_2_O_3_-NH_4_OH@SiO_2_(APTMS) nanoparticles (NPs).

The γ-Fe_2_O_3_-NH_4_OH@SiO_2_(APTMS) NPs were synthesized by co-precipitation of ferrous and ferric salts in a basic medium to form a magnetic core, stabilized in NH_4_OH medium, coated with silica, and functionalized by an amino group from (3-Aminopropyl) trimethoxysilane (APTMS), as shown in Figure 1.

### 2.2. Characterization of γ-Fe_2_O_3_-NH_4_OH@SiO_2_(APTMS) NPs

The synthesized γ-Fe_2_O_3_-NH_4_OH@SiO_2_(APTMS) NPs were characterized in order to perform a structural study, thermogravimetric analysis, morphological and compositional study, and surface area and charge study, and to determine the magnetic properties.

#### 2.2.1. Structural Study

**Figure 2:**
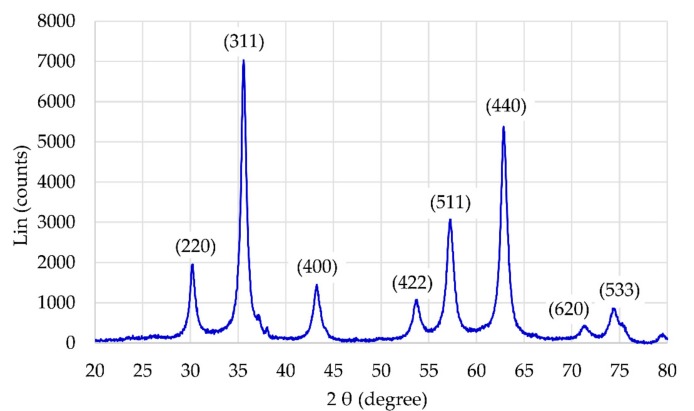
XRD of γ-Fe_2_O_3_ NPs.

The presence of the magnetic γ-Fe_2_O_3_ NP core of the particles was confirmed by X-ray diffraction (XRD). All diffraction peaks in Figure 2 are consistent with the standard structure of maghemite (JCPDS Card No.: 39-1346). By taking three of the most intensive peaks, corresponding to the crystal planes of (311), (440), and (511), the average crystalline size of the γ-Fe_2_O_3_ NPs is (19.1 ± 3.3) nm, as calculated by the Debye-Scherrer’s equation.

**Figure 3:**
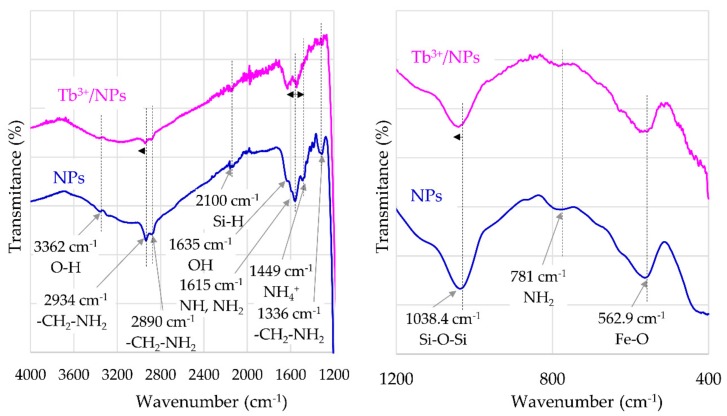
FT-IR spectra for γ-Fe_2_O_3_-NH_4_OH@SiO_2_(APTMS) NPs and Tb^3+^/γ-Fe_2_O_3_-NH_4_OH@SiO_2_(APTMS) NPs.

FT-IR spectra of γ-Fe_2_O_3_-NH_4_OH@SiO_2_(APTMS) NPs are presented in Figure 3. The characteristic peak at 3362 cm^−1^ indicates the stretching vibrations of O–H groups; the peaks at 2934 cm^−1^ and 2890 cm^−1^ correspond to the stretching vibration of –CH_2_–NH_2_ groups. The peak at 1635 cm^−1^ can be assigned to the OH groups. The peak at 1615 cm^−1^ indicates the scissoring vibrations of N–H in NH_2_ groups; meanwhile, the peak at 1449 cm^−1^ confirms the presence of NH_4_^+^ groups. The peak at 1336 cm^−1^ is significant for the wagging and twisting vibration of the –CH_2_–NH_2_ bond. The asymmetric stretching vibration of the Si–O–Si bond corresponds to the peak at 1038.4 cm^−1^. The absorption peak at 781 cm^−1^ corresponds to wagging and twisting of NH_2_ amino groups, and the absorption peak at 562.9 cm^−1^ corresponds to stretching vibrations of the Fe–O bond. Figure 3 shows the shifting of peaks, corresponding to characteristic groups of synthesized γ-Fe_2_O_3_-NH_4_OH@SiO_2_(APTMS) NPs, by adsorbed Tb^3+^. The peak at 2934 cm^−1^, which corresponds to stretching vibrations of –CH_2_–NH_2_ bonds for γ-Fe_2_O_3_-NH_4_OH@SiO_2_(APTMS) NPs, is shifted to the peak at 2941 cm^−1^ for Tb^3+^/γ-Fe_2_O_3_-NH_4_OH@SiO_2_(APTMS) NP systems. The peak at 1615 cm^−1^, corresponding to scissoring vibrations of N–HHHH in NH_2_ bonds for γ-Fe_2_O_3_-NH_4_OH@SiO_2_(APTMS) NPs, is shifted to 1636 cm^−1^ and to 1545 cm^−1^ for the Tb^3+^/γ-Fe_2_O_3_-NH_4_OH@SiO_2_(APTMS) NP system. For this system, a minor peak shift of about 2 cm^−1^ to a higher wavenumber (with respect to γ-Fe_2_O_3_-NH_4_OH@SiO_2_(APTMS) NPs) was observed. This corresponds to the asymmetric stretching vibration of the Si–O–Si bond.

The FT-IR spectra of γ-Fe_2_O_3_-NH_4_OH@SiO_2_(APTMS) NPs after adsorption of Tb^3+^ exhibit shifts, intensity variations, and broadening of the peaks. With respect to the investigation of Dubey and Gandhi [34], these modifications in FT-IR spectra confirm the presence of contact between the NPs and Tb^3+^, as shown in Figure 3.

The amount 0.0448 mmol/g of amino groups on the γ-Fe_2_O_3_-NH_4_OH@SiO_2_(APTMS) NPs, obtained with potentiometric titration, confirms successful functionalization with amino groups [35].

#### 2.2.2. Thermogravimetric Analysis

**Figure 4:**
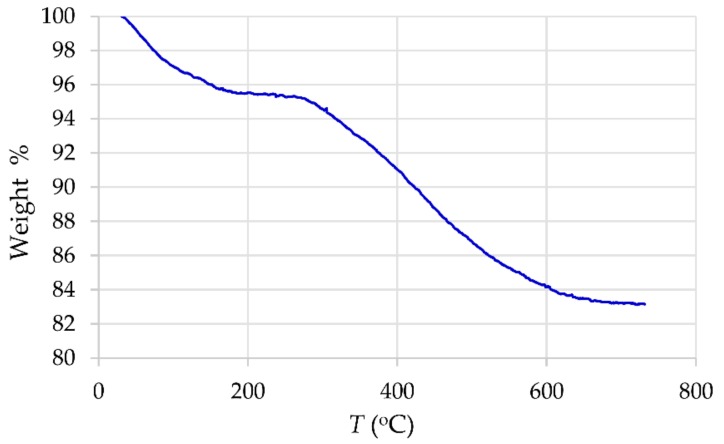
Thermogravimetric analysis (TGA) for γ-Fe_2_O_3_-NH_4_OH@SiO_2_(APTMS) NPs.

The results of thermogravimetric analysis (TGA) are shown in Figure 4, where the mass loss of γ-Fe_2_O_3_-NH_4_OH@SiO_2_(APTMS) NPs by heating up to 800 °C is presented. The TGA curve shows that the weight loss occurs in two steps. The first step, probably related to the evaporation of absorbed moisture, can be observed in the range from 25 °C to 180 °C and leads to a loss of 4.7%. The second decomposition step occurs in the interval from 280 °C to 800 °C after a plateau where the material is thermally stable. This step corresponds to a weight loss of 12.1% and is associated with thermal destruction of amino NH_2_ groups and removal of the alkyl chains from the Si–O–Si framework (cracking of the residual surface alkyl siloxane Si–O–Si species) [36,37,38].

#### 2.2.3. Morphological and Compositional Study

**Figure 5:**
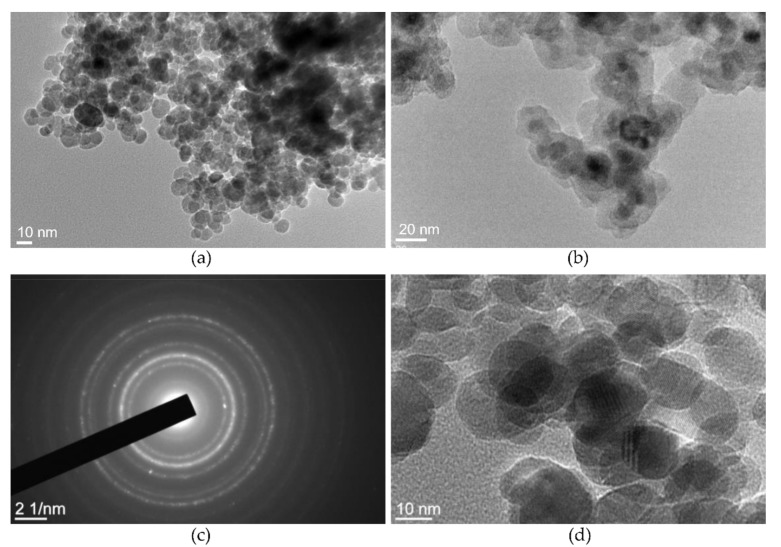
TEM of γ-Fe_2_O_3_ NPs (**a**), γ-Fe_2_O_3_-NH_4_OH@SiO_2_(APTMS) NPs (**b**), the electron diffraction pattern of γ-Fe_2_O_3_ NPs (**c**), the spinel structure of γ-Fe_2_O_3_ NPs (**d**).

The TEM photos in Figure 5 display the morphology of the γ-Fe_2_O_3_ NPs and γ-Fe_2_O_3_-NH_4_OH@SiO_2_(APTMS) NPs. Figure 5a shows the relatively spherical morphology of the maghemite NPs with the narrow particle size distribution of (13.7 ± 4.1) nm. The TEM images in Figure 5b show the relatively spherical morphological structure of γ-Fe_2_O_3_-NH_4_OH@SiO_2_(APTMS) NPs samples with an eminent surface layer of amorphous siloxane (SiO_2_). The average size of γ-Fe_2_O_3_-NH_4_OH@SiO_2_(APTMS) NPs is about 55 nm. The electron diffraction pattern of the γ-Fe_2_O_3_ sample in Figure 5c confirms the formation of a spinel crystal structure with a relatively high material crystallinity, which is consistent with the HRTEM image of γ-Fe_2_O_3_ NPs in Figure 5d.

**Figure 6:**
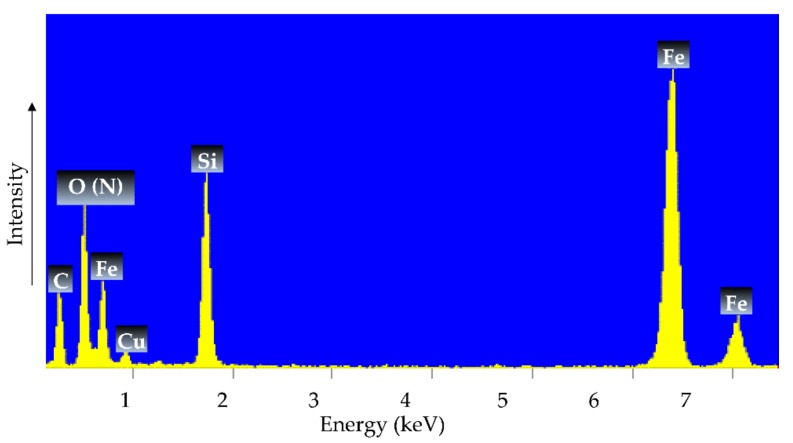
EDXS of the new, gradually synthesized γ-Fe_2_O_3_-(NH_4_OH)@SiO_2_(APTMS) NPs.

**Figure 7:**
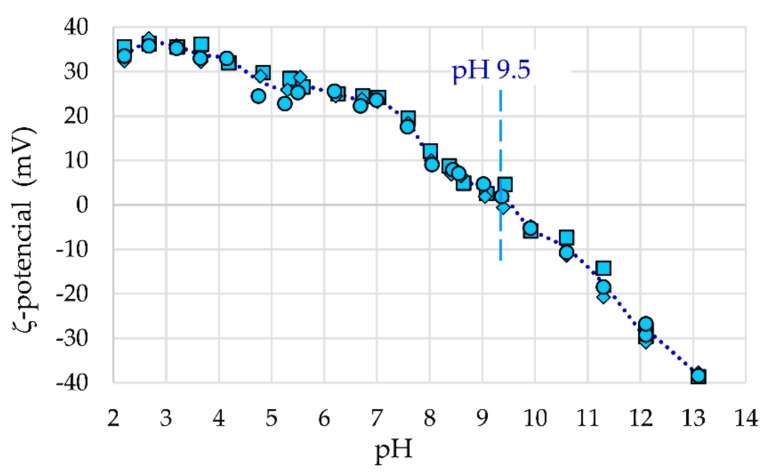
The ζ-potential for γ-Fe_2_O_3_-(NH_4_OH)@SiO_2_(APTMS) NPs.

Figure 6 shows the EDXS spectrum of γ-Fe_2_O_3_-NH_4_OH@SiO_2_(APTMS) NPs, where the presence of Fe, O (N), Si, C, and Cu is evident. The elementary composition confirms the success of the synthesis of a magnetic maghemite core, a silicate shell, and functionalization with amino groups. The EDXS analysis of the sample indicated large peaks indicating iron (Fe) and silicon (Si), characteristic of Fe–O bond formation and siloxane (Si–O–Si) bonds, respectively, while there is overlap between the elemental peaks of oxygen (O) and nitrogen (N). Note that the peaks of Cu and C in the spectra belong to the TEM’s copper-grid-supported transparent carbon foil; the sample was measured on this grid during the EDXS analysis [39,40]. From the obtained EDXS pattern, it can be concluded that the amino groups of the APTMS are successfully attached to the surface of the γ-Fe_2_O_3_-NH_4_OH@SiO_2_, which is in a good agreement with the ζ-potential, as shown in Figure 7.

#### 2.2.4. Surface Area and Charge Study

Brunauer-Emmet-Teller (BET) analysis gives a specific surface area of 6.41 m^2^/g for γ-Fe_2_O_3_-(NH_4_OH)@SiO_2_(APTMS) NPs. At Barrett-Joyner-Halenda (BJH) adsorption, the average pore size is 5.4 nm with a total pore volume of 0.0505 cm^3^/g. Meanwhile, at BJH desorption, the average pore size is 5.7 nm with a total pore volume of 0.05309 cm^3^/g. According to the BET specific surface area at a relative pressure of 0.3, the predicted average γ-Fe_2_O_3_-(NH_4_OH)@SiO_2_(APTMS) NP size is 53.6 nm.

In Figure 7, the ζ-potential as a function of pH of the synthesized γ-Fe_2_O_3_-NH_4_OH@SiO_2_(APTMS) NPs is shown. It can be seen that the isoelectric point is equal to 9.5, which confirms functionalization with amino groups from APTMS. The magnetic stabilized and functionalized core@shell NPs already show stability at pH 7, while they are fully stable at pH < 4.5, where the ζ-potential is >30 mV, and at pH > 12, where the ζ-potential is <−30 mV.

#### 2.2.5. Magnetic Study

**Figure 8:**
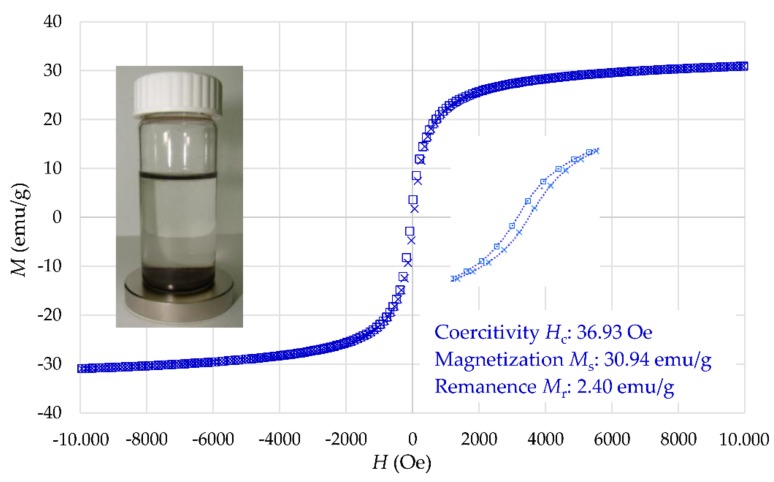
Magnetization curve of γ-Fe_2_O_3_-NH_4_OH@SiO_2_(APTMS) NPs.

Figure 8 displays the magnetization curve of γ-Fe_2_O_3_-NH_4_OH@SiO_2_(APTMS) NPs at room temperature, where the *M_S_* value is 30.94 emu/g. However, this value of *M_S_* is similar to that of maghemite NPs [41,42]. These NPs show close superparamagnetic behavior with smaller remanence magnetization and smaller coercitivity than γ-Fe_2_O_3_ NPs. The obtained magnetization properties confirm simple separation of Tb^3+^/γ-Fe_2_O_3_-NH_4_OH@SiO_2_(APTMS) NPs from aqueous solution.

### 2.3. Adsorption Study

**Figure 9:**
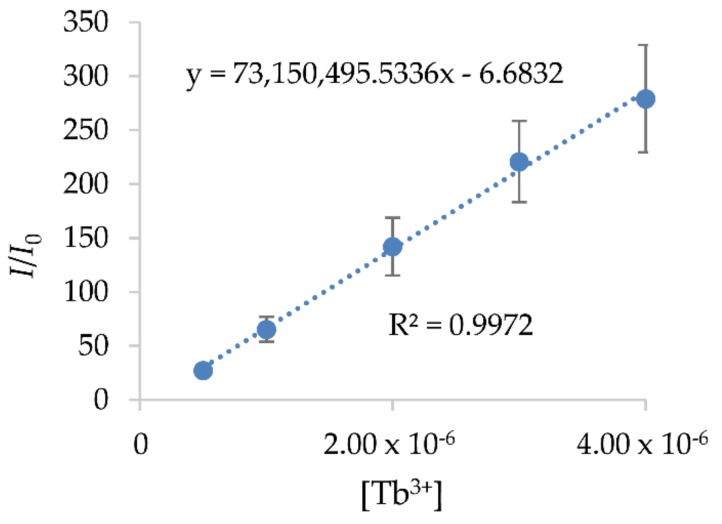
Calibration curves of Tb^3^ adsorption from the fluorescence spectrometer.

The absorption wavelength of the prepared standard solutions, obtained by UV/VIS spectroscopy, is 312 nm. The calibration curves of the prepared standard solutions with a low concentration of Tb^3+^, obtained by a Perkin Elmer LS 55 fluorescence spectrometer, are given in Figure 9. The correlation coefficient of the calibration curves is higher than 0.99.

#### 2.3.1. NP Mass Influence

**Figure 10:**
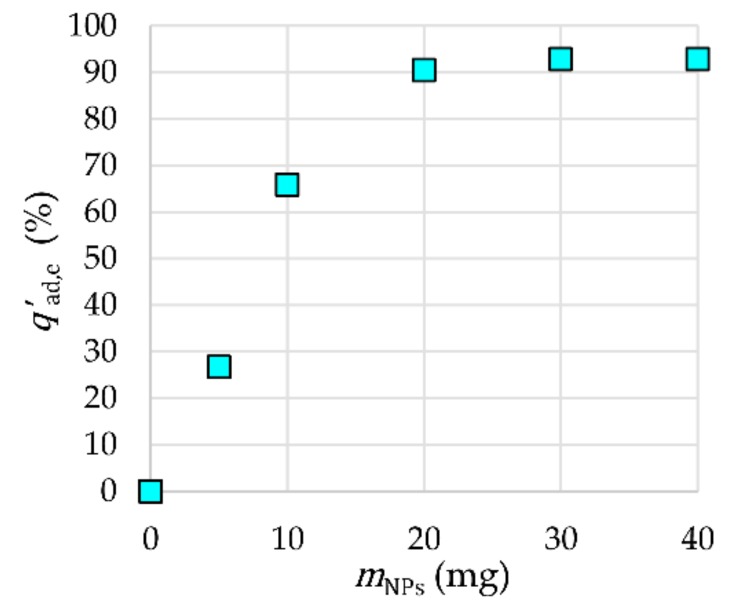
Influence of the mass of NPs on adsorption efficiency.

The influence of the mass of γ-Fe_2_O_3_-NH_4_OH@SiO_2_(APTMS) NPs on the adsorption efficiency is presented in Figure 10. The equilibrium mass *m*_NPs,eq_ of γ-Fe_2_O_3_-NH_4_OH@SiO_2_(APTMS) NPs as adsorbent for Tb^3+^, with respect to the adsorption efficiency, was 30 mg for a solution volume *V* = 20 mL, *t*_ad_ = 120 min, *c*_ads,0_ = 2 × 10^−6^ M, and *T*_ad_ = 25 °C, where the reached adsorption efficiency was 93%. An ultrasonic bath was used to mix the samples at pH 7. Figure 10 shows that the adsorption efficiency increased significantly by increasing the mass of NPs up to 30 mg due to an increase in the number of adsorption sites. Further increasing of the mass of NPs does not influence the adsorption efficiency; therefore, 30 mg of NPs was taken as *m*_NPs,eq_ (i.e., an adsorbent dosage of 1.5 mg/mL) for further investigation of the adsorption process. This value offers a good compromise between cost and efficiency.

#### 2.3.2. Kinetic Study

**Figure 11:**
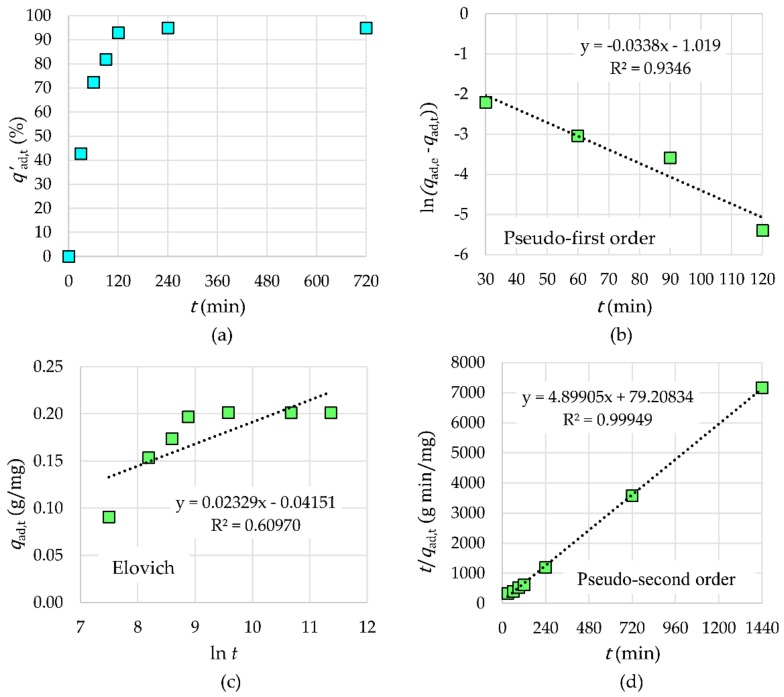
The influence of time on adsorption efficiency (**a**) and kinetic models for Tb^3+^ adsorption: pseudo-first order (**b**), Elovich (**c**), and pseudo-second order (**d**).

The adsorption kinetics of Tb^3+^ by γ-Fe_2_O_3_-NH_4_OH@SiO_2_(APTMS) NPs is shown in Figure 11. One can see that Tb^3+^ were completely removed from the aqueous solution within 120 min of contact time (*V* = 20 mL, *m*_NPs_ = 30 mg, *c*_ads,0_ = 2 × 10^−6^ M, and *T*_ad_ = 25 °C). Among the tested models (pseudo-first order, Elovich, and pseudo-second order), the experimental data fitted very well with the pseudo-second order rate equation with an *R*^2^ value close to 0.9995. The appropriate values of parameters of the pseudo-second order kinetic model are as follows: *k*_2_ = 0.3030 g·mg^−1^·min^−1^ and *q*_ad,e_ = 0.2041 mg/g for Tb^3+^ adsorption. The rate-limiting step may be chemical adsorption or chemisorption through sharing or exchange of electrons between adsorbents and adsorbates. In the initial time interval of 120 min, the adsorption efficiency increases rapidly, which means that the chemical adsorption worked at a low rate.

#### 2.3.3. Adsorption Isotherms

**Figure 12:**
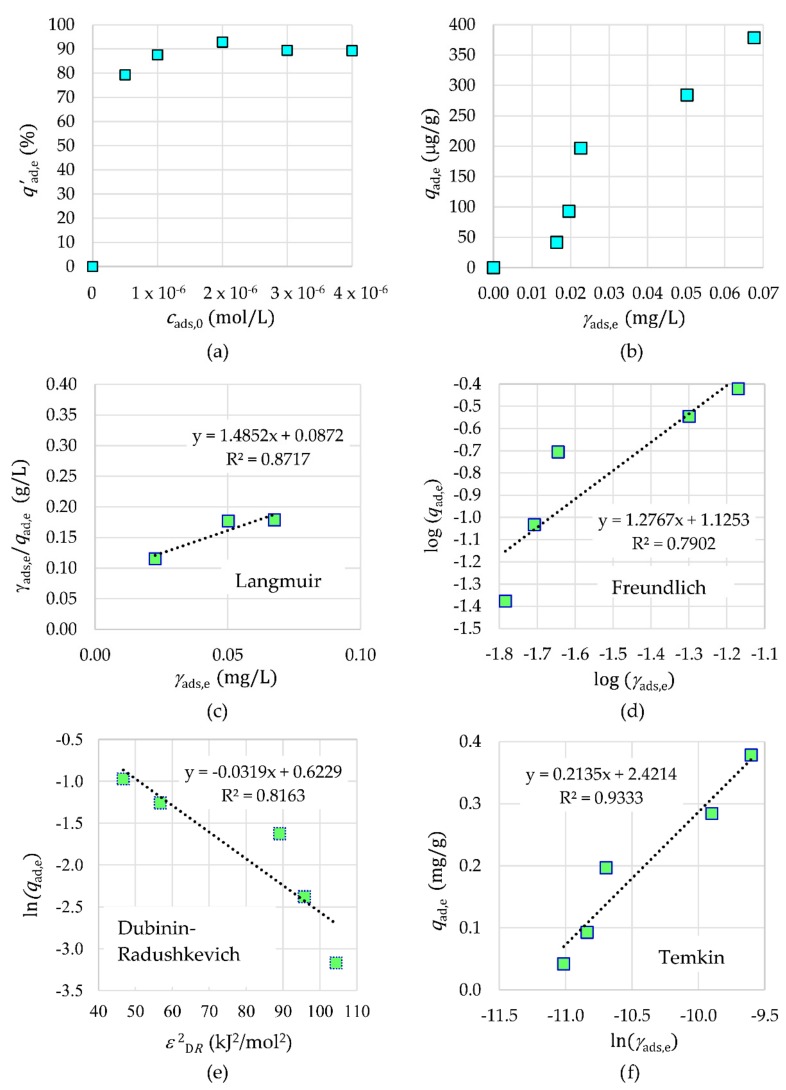
The influence of initial concentration (**a**) and equivalent concentration (**b**) on adsorption. Adsorption isotherms for Tb^3+^ on γ-Fe_2_O_3_-NH_4_OH@SiO_2_(APTMS) NPs: Langmuir (**c**), Freundlich (**d**), Dubinin–Radushkevich (**e**), and Temkin (**f**).

The results in Figure 12 show that the adsorption efficiency increased with an increasing initial concentration of Tb^3+^ *c*_ads,0_ and reached a plateau at a higher concentration due to saturation of the number of binding sites in a fixed amount of NPs. The optimum initial concentration relates mainly to the availability of active sites, which is interrelated to the presence of surface functional groups. The adsorption capacity in equilibrium *q*_ads,e_ for Tb^3+^ with respect to the equilibrium mass concentration of adsorbate *γ*_ads,e_ shows an S-type adsorption isotherm (Figure 12). This means low adsorption at a very low Tb^3+^ concentration and then a rapid increase of adsorption with the increasing Tb^3+^ concentration, until there is no more space on the surface of the nanoparticle to hold the adsorbates. The S-type isotherm confirms that the synthesized NPs are a porous material with the cohesive forces between adsorbate and adsorbent being greater than those between adsorbate molecules. To characterize the adsorption equilibria, the linear data from Langmuir, Freundlich, Dubinin–Radushkevich, and Temkin isotherms for Tb^3+^ as adsorbate are presented in Figure 12. The experimental data fitted very well with the Temkin adsorption isotherm with an *R*^2^ value of 0.9333 for Tb^3+^. The values of appropriate parameters of the Temkin isotherm are as follows: *b*_T_ = 1.1605 × 10^4^ J·g·mol^−1^·mg^−1^ and *A*_T_ = 8.4242 × 10^4^ L/g for Tb^3+^. The Temkin isotherms confirm the chemisorption and the assumptions that the adsorption energy decreases linearly with the increase in coverage of the adsorbent surface and that the binding energy is uniformly distributed.

Due to the fact that the Temkin isotherm model fits very well with the Tb^3+^ adsorption data, it can be concluded that the heat of adsorption of all molecules of the tested Tb^3+^ in the γ-Fe_2_O_3_-NH_4_OH@SiO_2_(APTMS) NP layer is not the same. Furthermore, the adsorption is not monolayer, and transmigration of adsorbate in the plane of the NP surface is present.

The maximum adsorption capacity relates mainly to the availability of an active site, which is correlated to the presence of surface functional groups. The neutral nitrogen atoms of –NH_2_ and –NH– groups have a lone pair of electrons that could be shared with rare earth ions, thus leading to the formation of a coordination bond between the N atoms of the amino groups from APTMS and rare earth atoms. The coordination number of Tb^3+^ is 9. Due to the hypothesis that the primary and secondary amino groups are the most important sites for Tb^3+^ adsorption, the predicted adsorption mechanism is based on coordination of Tb^3+^ with one or two amino groups. Meanwhile, the remaining coordination sites may be provided by water molecules [43]. The predicted adsorption mechanism for Tb^3+^ adsorption on the surface of γ-Fe_2_O_3_-NH_4_OH@SiO_2_(APTMS) NPs can be written as:

R-NH_2_ + Tb^3+^ ⇌ [Tb(R-NH_2_)(H_2_O)_8_]^3+^. (1)

#### 2.3.4. Thermodynamic Study

**Figure 13:**
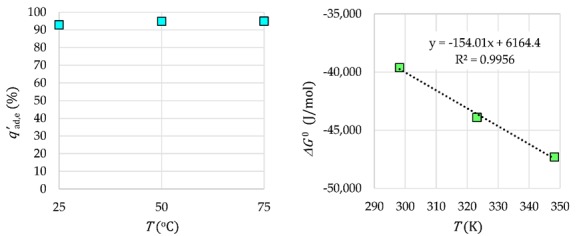
The influence of temperature and thermodynamics for Tb^3+^ adsorption.

The Tb^3+^ adsorption efficiency on γ-Fe_2_O_3_-NH_4_OH@SiO_2_(APTMS) NPs at various temperatures and the linearization of the Van’t Hoff equation are presented in Figure 13. The thermodynamic data are given in Table 1.

In the case of Tb^3+^ adsorption on γ-Fe_2_O_3_-NH_4_OH@SiO_2_(APTMS) NPs, a change in the standard enthalpy or the adsorption heat Δ*H*^0^ > 0 is obtained. Therefore, this adsorption is an endothermic reaction. The resulting positive change in the standard entropy Δ*S*^0^ > 0, similar to the negative Gibbs free energy Δ*G*^0^ < 0, indicates a spontaneous adsorption reaction. The endothermic and spontaneous adsorption reaction of Tb^3+^ on γ-Fe_2_O_3_-NH_4_OH@SiO_2_(APTMS) NPs can be explained by good dissolution of Tb^3+^ in water. When dissolving Tb-salt in water, water molecules coat the ions, and molecular bonds between the ions and water are formed: hydration. In order for Tb^3+^ to be adsorbed, they must lose a part of their hydrating coat. This ion dehydration process requires energy that is greater than the released heat during adsorption of the ions on the surface of the adsorbent. Therefore, the removal of water from the Tb^3+^ coating is an endothermic process whose energy exceeds the energy of the exothermic reaction of adsorption of ions that are attached to the surface. The obtained Gibbs energy values are negative, which is expected for a spontaneous adsorption process.

## 3. Materials and Methods

### 3.1. Materials

For each used material, its chemical formula, purity, molar mass, density at 25 °C, and Chemical Abstract Service (CAS) number and supplier are given as follows: 2-propanol (C_3_H_8_O, 99.8%, 60.1 g/mol, 0.785 g/mL, CAS 67-63-0, Gram Mol), 3-aminopropyltrimethoxysilane (APTMS) (C_6_H_17_NO_3_Si, 97%, 179.29 g/mol, 1.027 g/mL, CAS 13822-56-5, Merck), ammonium hydroxide (NH_4_OH, 25%, 35.05 g/mol, 0.91 g/mL, CAS 1336-21-6, Gram Mol), ethanol (C_2_H_5_OH, 96%, 46.07 g/mol, 0.810 g/mL, CAS 64-17-5, Honeywell), ferric chloride hexahydrate (FeCl_3_·6H_2_O, 270.3 g/mol, 1.82 g/mL, CAS 10025-77-1, Sigma Aldrich), ferrous chloride tetrahydrate (FeCl_2_·4H_2_O, 198.81 g/mol, 1.93 g/mL, CAS 13478-10-9, Sigma Aldrich), terbium(III)chloride hexahydrate (TbCl_3_·6H_2_O, 99.9%, 373.38 g/mol, 4.35 g/mL, CAS 13798-24-8, Sigma Aldrich), tetraethyl orthosilicate (TEOS) (C_6_H_20_O_4_Si, 99%, 208.33 g/mol, 0.94 g/mL, CAS 78-10-4, Sigma Aldrich), tiron monohydrate (C_6_H_4_Na_2_O_8_S_2_·H_2_O, 98%, 314.2 g/mol, CAS 149-45-1, Sigma Aldrich), tris(hydroxymethyl) aminomethane (H_2_NC(CH_2_OH)_3_, 121.14 g/mol, 0.8 g/mL, CAS 77-86-1, Merck). All chemicals were used as received without further purification. For the preparation of all suspensions and solutions, deionized water (dH_2_O) was used.

### 3.2. Synthesis of γ-Fe_2_O_3_-NH_4_OH@SiO_2_(APTMS) NPs

At first, 25% NH_4_OH (150 mL) was heated up to (87 ± 2) °C and stirred (300 rpm). The solution (50 mL of 0.5 M) of FeCl_2_⋅4H_2_O (1.6735 g) and FeCl_3_⋅6H_2_O (4.5505 g) was added to the prepared ammonium solution and heated by reflux at (87 ± 2) °C. The reaction (60 min, pH 9.9) was carried out in a round-bottom reaction flask. The particles were precipitated and separated from the reaction mixture by a permanent magnet and washed several times with dH_2_O. The washing process includes addition of dH_2_O, decantation of NPs by a magnet, and removal of the supernatant. The 25% NH_4_OH solution (25 mL) was added to the washing sediment of γ-Fe_2_O_3_ NPs (~1.6 g) into the reaction flask. After the reaction (24 h, 50 °C) under continuous stirring, the stabilized γ-Fe_2_O_3_-NH_4_OH NPs were separated from the supernatant with a permanent external magnet and the dH_2_O was added. The silica shell from TEOS and functionalization with APTMS was performed “in-situ”. For this purpose, 2-propanol (66 mL), dH_2_O (15.42 mL), 25% NH_4_OH solution (1.7 mL), the prepared γ-Fe_2_O_3_-NH_4_OH colloid suspension (4.93 mL, 1.038 g/mL), TEOS (0.324 mL), and APTMS (0.518 mL) were mixed under magnetic stirring (500 rpm) in a closed vessel (24 h, 25 °C). After the completed reaction, the sediment was washed with ethanol and dH_2_O several times, and the magnetic stabilized and functionalized γ-Fe_2_O_3_-NH_4_OH@SiO_2_(APTMS) core@shell NPs were decanted by using an external magnet.

### 3.3. Characterization and Adsorption Techniques

The synthesized γ-Fe_2_O_3_-NH_4_OH@SiO_2_(APTMS) NPs were characterized in order to analyze their structural, morphological, thermogravimetric, surface charge, and magnetic properties. XRD analysis was used for structural characterization with a Brucker D4 Endavor X-ray diffractometer, equipped with Cu Kα radiation (*λ* = 0.15418 nm); the measurement was done at room temperature within the range of Bragg’s angle 2θ from 20° to 80°. A Fourier Transform Infrared (FT-IR) spectrometer of the type PerkinElmer with Spectrum GX software was applied to qualitatively identify the functional groups on the material. FT-IR spectra were recorded at room temperature in a range of 400–4000 cm^−1^ in the transmittance mode. The amounts of the functional groups were quantitatively determined via a potentiometric titration using a two-burette Mettler T-70 instrument, equipped with a Mettler T DG 117 combined glass electrode. The burettes were filled with 0.1 M HCl and 0.1 M KOH. The stability criterion for taking a reading after each addition was set to 0.1 mV/20 s, where 20 s was the minimum time to reach equilibrium conditions between additions of the titrant, and the maximum time was set to 180 s. Thermogravimetric analysis (TGA) was performed on the dried powdered samples (previously dried at 80 °C for 10 h) in alumina crucibles, with the heating rate of 20 °C/min by using a PerkinElmer TGA 4000 under a nitrogen atmosphere (20 mL/min) up to 900 °C. The particles were observed by transmission electron microscopy (TEM) using a Jeol JEM-2100 (JEOL USA, Inc., Peabody, MA, USA), where the nanoparticles were deposited on a copper-grid-supported, perforated, and transparent carbon film. Energy Dispersive X-ray Spectroscopy (EDXS) mapping analysis was performed at 200 kV using a JEOL-2010 microscope. The Brunauer–Emmet–Teller (BET) technique was used to determine the specific surface area of nanoparticles by using a Micromeritics Flow Prep 060 with a Tristar II 3020. All samples were degassed at 110 °C for 24 h prior to each measurement. The specific surface area was evaluated in the 0.05–0.3 range of relative pressure in nitrogen gas at a temperature of 77.35 K after 24 h. To determine the electro kinetic effects at a moving interface, Dynamic Light Scattering (DLS) analysis was used, where the ζ-potential was measured by ZetaSizer Nano series Malvern Instruments. The saturation magnetization of NPs was studied by a Vibrating Sample Magnetometer (VSM, Lake Shore 7400) at a magnetic field *H* of 10,000 Oe and room temperature. The excitation wavelength of fluorescence spectroscopy was determined by a Perkin Elmer 35 UV/VIS spectrophotometer. To determine the amount of adsorbed Tb^3+^ on the surfaces of NPs, the emission wavelengths were identified using a Perkin Elmer LS 55 fluorescence spectrometer. In order to increase the intensity of Tb^3+^ fluorescence, complexes of Tb^3+^ with tiron monohydrate as a ligand were used. The calibration curve was determined with standard solutions, prepared from 10^−4^ M Tb^3+^ solution, 10^−4^ M C_6_H_4_Na_2_O_8_S_2_·H_2_O solution, and 0.05 M tris(hydroxymethyl) aminomethane solution at a pH of 7 and a temperature of 25 °C. The measurements of fluorescence of Tb^3+^ complexes were performed with sample solutions, where, instead of 10^−4^ M Tb^3+^ solution, the same volume of supernatant (after removal of adsorbed Tb^3+^ on NPs) was used.

### 3.4. Procedure for Adsorption

The adsorption study was performed by mixing the prescribed mass of γ-Fe_2_O_3_-NH_4_OH@SiO_2_(APTMS) NPs (*m*_NPs_) with a water solution of initial Tb^3+^ concentrations (*c*_ads,0_) at a prescribed temperature (*T*_ad_) and at a prescribed adsorption time (*t*_ad_). The adsorption capacity of the mass (mg) of adsorbed Tb^3+^ per mass (g) of NPs *q*_ad_ (mg/g) and adsorption efficiency *q*’_ad_ (%) were calculated by using Equations (2) and (3):

(2) $$q_{\mathrm{ad}}\;=\;\frac{\left(c_{\mathrm{ads},0}\;-\;c_{\mathrm{ads},e}\right)M_{\mathrm{ads}}V}{m_{\mathrm{NPs}}}$$

(3) $$q\prime_{\mathrm{ad}}\;=\;\frac{c_{\mathrm{ads},0}\;-\;c_{\mathrm{ads},e}}{c_{\mathrm{ads},0}}$$

where *c*_ads,0_ (mol/L) and *c*_ads,e_ (mol/L) are the initial and equilibrium concentrations of Tb^3+^, respectively, *V* (L) denotes the solution volume, *M*_ads_ (g/mol) is the molar mass of the adsorbate, and *m*_NPs_ (g) is the mass of NPs.

To determine the affinity of Tb^3+^ on the γ-Fe_2_O_3_-NH_4_OH@SiO_2_(APTMS) NP surface, the adsorption conditions (equilibrium mass of adsorbent, adsorption time, initial Tb^3+^ concentration, and temperature of adsorption) were changed. The Tb^3+^/γ-Fe_2_O_3_-NH_4_OH@SiO_2_(APTMS) NPs were removed from aqueous solution with an external permanent magnet. For determination of the adsorption capacity, UV/VIS spectroscopy and fluorescence spectroscopy were used.

The influence of the equilibrium mass *m*_NPs,eq_ of γ-Fe_2_O_3_-NH_4_OH@SiO_2_(APTMS) NPs as an adsorbent up to 40 mg (to weigh a mass, the Mettler Toledo XPE205 balance was used) for Tb^3+^ on the adsorption was tested for: *V* = 20 mL, *t*_ad_ = 120 min, *c*_ads,0_ = 2 × 10^−6^ M, and *T*_ad_ = 25 °C.

Then, the influence of adsorption time (up to 1440 min) at the determined *m*_NPs,eq_ for Tb^3+^, *V* = 20 mL, *c*_ads,0_ = 2 × 10^−6^ M, and *T*_ad_ = 25 °C was investigated. The equivalence adsorption time *t*_ad,eq_ and the most appropriate kinetic model were determined. The tested kinetic models were pseudo-first order, Elovich, and pseudo-second order models using the equations shown in Table 2, where the symbols used are: *q*_ad,t_ (mg/g), adsorption capacity in time *t*; *q*_ad,e_ (mg·g^−1^·min^−1^), equilibrium adsorption capacity; *t* (min), adsorption time; *q*_ad,e,exp_ (mg/g), experimentally obtained equilibrium adsorption capacity; *k*_1_ min^−1^, a constant of the pseudo-first order model; α_E_ (mg·g^−1^·s^−1^), the initial sorption rate in the Elovich model; *β*_E_ (g/mg), a constant related to the extent of surface coverage and activation energy for chemisorption in the Elovich model; and *k*_2_ (g·mg^−1^·min^−1^), a constant of the pseudo-second order model [44,45]. In the pseudo-first order kinetic model, the appearance of adsorption is assumed only on localized sites. Further assumptions are: no interaction between adsorbate ions, independence of adsorption energy on surface coverage, and the influence of the saturated monolayer of adsorbates on the NP surface on maximum adsorption. On the other hand, the Elovich model considers the interaction between adsorbate ions by adsorption on localized sites of NPs and a linear increase of the adsorption energy with the surface coverage. The pseudo-second order kinetic model is based on the assumption that the rate-limiting step may be chemical adsorption involving valence forces through sharing or exchange of electrons between the adsorbent and adsorbate, where the removal from a solution is due to the physicochemical interaction between two phases.

The initial Tb^3+^ concentration was varied (up to 4 × 10^−6^ M) in order to study the adsorption isotherm of Tb^3+^ on the surface of γ-Fe_2_O_3_-NH_4_OH@SiO_2_(APTMS) NPs at *V* = 20 mL, *m*_NPs,eq_, *t*_ad,eq_, and *T*_ad_ = 25 °C. Low Tb^3+^ concentrations were used due to investigation of the adsorption of Tb^3+^ from aqueous solution with a very low concentration of Tb^3+^ (from 5 × 10^−7^ M to 4 × 10^−6^ M). The experimental data were interpreted by Langmuir, Freundlich, Dubini–Radushkevich, and Temkin adsorption isotherms using the equations shown in Table 3, where the symbols used are: *q*_ad,e_ (mg/g), equilibrium adsorption capacity; *q*_m_ (mg/g), adsorption monolayer coverage capacity; *K*_L_ (L/mg), Langmuir isotherm constant; *γ*_ads,e_ (mg/L), equilibrium mass concentration of adsorbate; *K*_F_ ((mg/g)·(L/mg)^1/*n*^), Freundlich isotherm constant; *n*(/), dsorption intensivity; *q*_s_ (mg/g), theoretical isotherm saturation capacity; *K*_DR_ (mol^2^·kJ^−2^), *ε*_DR_ (kJ^2^·mol^−2^), Dubinin–Radushkevich constant; *T* (K), temperature; *R* (8.314 J·mol^−1^·K^−1^), a gas constant; *b*_T_ (J·g·mol^−1^·mg^−1^), Temkin isotherm constant; *A*_T_ (L/g), the Temkin isotherm equilibrium binding constant [46,47,48,49,50]. The Langmuir isotherm describes quantitatively the formation of a monolayer adsorbate on the outer surface of NPs, and after that no further adsorption takes place. The Langmuir isotherm is valid for monolayer adsorption onto a surface containing a finite number of identical sites. The model assumes uniform energies of adsorption onto the surface and no transmigration of the adsorbate in the plane of the surface. The Freundlich isotherm describes the adsorption characteristics of a heterogeneous surface. The Dubinin–Radushkevich isotherm model expresses the adsorption mechanism with a Gaussian energy distribution onto a heterogeneous surface of NPs. The Temkin isotherm model assumes that the heat of adsorption of all molecules in the layer decreases linearly rather than logarithmically with coverage, and it takes into account the adsorbate–adsorbent interaction.

The thermodynamic analysis of Tb^3+^ adsorption on the surface of γ-Fe_2_O_3_-NH_4_OH@SiO_2_(APTMS) NPs was done at various temperatures of adsorption. The free energy Δ*G*^0^ was evaluated by applying Gibb’s free energy (Equation (4)) and the standard enthalpy change Δ*H*^0^; entropy change Δ*S*^0^ was calculated from the Van’t Hoff Equation (5):

(4) $$∆G^{0}=-R\cdot T\cdot \ln K_{d}=-R\cdot T\cdot \ln\left(\gamma_{r}\cdot \frac{q_{\mathrm{ad},e}}{\gamma_{\mathrm{ads},e}}\right)$$

(5) $$∆G^{0}=∆H^{0}-T\cdot ∆S^{0}$$

where Δ*G*^0^ (J/mol) denotes the Gibb’s energy change, Δ*H*^0^ (J/mol) is the standard enthalpy change, Δ*S*^0^ (J·mol^−1^·K^−1^) is the entropy change, *T* (K) is the adsorption temperature, *R* (8.314 J·mol^−1^·K^−1^) is the gas constant, *k*_d_ (/) is the distribution coefficient, *γ*_r_ (1000 g/L) is the mass concentration of water, *q*_ad,e_ (mg/g) is the equilibrium mass concentration, and *γ*_ads,e_ (mg/L) is the equilibrium mass concentration of the adsorbate in aqueous solution.

On the basis of the calculated distribution constant *K*_d_ and Gibb’s energy Δ*G*^0^ at various temperatures and from the linearization of Equation (3) by a linear plot of Δ*G*^0^ = *f*(*T*), the standard enthalpy change Δ*H*^0^ and the entropy change Δ*S*^0^ were determined.

## 4. Conclusions

Magnetic γ-Fe_2_O_3_ NPs stabilized with NH_4_OH, coated by SiO_2_, and functionalized by APTMS were synthesized, characterized, and investigated as an adsorbent for Tb^3+^ adsorption from aqueous solution. One of the advantages of γ-Fe_2_O_3_-NH_4_OH@SiO_2_(APTMS) NPs is their simple synthesis procedure and good properties, confirmed by XRD, FT-IR, TGA, TEM, EDXS, DLS, and VSM characterization and the adsorption process. The experimental data of endothermic spontaneous adsorption of Tb^3+^ were well-fitted to the Temkin’s adsorption isotherm and pseudo-second order kinetic model. The grafted and functionalized NPs have good magnetization properties. Therefore, NPs with adsorbed Tb^3+^ can be easily retrieved from the solution using a small external magnet. The main advantage of this study is that adsorption takes place at a neutral pH, which is environmentally friendly. Furthermore, the obtained high adsorption efficiency of the low concentration of Tb^3+^ from aqueous solution confirms a promising application of γ-Fe_2_O_3_-NH_4_OH@SiO_2_(APTMS) NPs as an adsorbent for Tb^3+^. The maximum adsorption efficiency was obtained at a contact time of 120 min, an adsorbent dosage of 1.5 mg/mL, an initial concentration of Tb^3+^ of 2 × 10^−6^ M, and at a temperature of 25 °C. In future work, it might be reasonable to investigate the optimal adsorption conditions (pH, agitation speed), the possibility of adsorption of other REE^3+^ on these functionalized magnetic nanoparticles, as well as the separation of various REE^3+^ by selective uptake.

## Figures and Tables

**Table 1:** Thermodynamic data.

Tb^3+^/NPs	Temperature (°C)		
	25	50	75
Δ*G*^0^ (J/mol)	−39,606	−43,900	−47,307
Δ*H*^0^ (J/mol)	6164	6164	6164
Δ*S*^0^ (J/(mol·K))	154	154	154

**Table 2:** Kinetic models for adsorption.

Kinetic Model	Nonlinear Form	Linear Form	Linear Plot
Pseudo-first order	$q_{\mathrm{ad},t}=\;q_{\mathrm{ad},e}\cdot \left(1-e^{-k_{1}\cdot t}\right)$	$\ln\left(q_{\mathrm{ad},e}-q_{\mathrm{ad},t}\right)=-k_{1}t+\ln q_{\mathrm{ad},e}$	$\ln\left(q_{\mathrm{ad},e,\exp}-q_{\mathrm{ad},t}\right)=f\left(t\right)$
Elovich	$q_{\mathrm{ad},t}=\frac{1}{\beta_{E}}\cdot \ln\left(\alpha_{E}\cdot \beta_{E}\cdot t\right)$	$q_{\mathrm{ad},t}=\frac{1}{\beta_{E}}\cdot \ln\left(\alpha_{E}\cdot \beta_{E}\right)+\frac{1}{\beta_{E}}\ln t$	$q_{\mathrm{ad},t}=\;f\left(\ln t\right)$
Pseudo-second order	$q_{\mathrm{ad},t}=\frac{k_{2}\cdot q_{\mathrm{ad},e}^{2}\cdot t}{1+k_{2}\cdot q_{\mathrm{ad},e}\cdot t}$	$\frac{t}{q_{\mathrm{ad},t}}=\frac{1}{q_{\mathrm{ad},e}}\cdot t+\frac{1}{k_{2}\cdot q_{\mathrm{ad},e}^{2}}$	$\frac{t}{q_{\mathrm{ad},t}}=f\left(t\right)$

**Table 3:** Adsorption isotherm models.

Adsorption Isotherm	Nonlinear Form	Linear Form	Linear Plot
Langmuir	$q_{\mathrm{ad},e}=\frac{q_{m}\cdot K_{L}\cdot \gamma_{\mathrm{ads},e}}{1+K_{L}\cdot \gamma_{\mathrm{ads},e}}$	$\frac{\gamma_{\mathrm{ads},e}}{q_{\mathrm{ad},e}}=\frac{1}{q_{m}}\cdot \gamma_{\mathrm{ads},e}\;+\;\frac{1}{q_{m}\cdot K_{L}}$	$\frac{\gamma_{\mathrm{ads},e}}{q_{\mathrm{ad},e}}=f\left(\gamma_{\mathrm{ads},e}\right)$
Freundlich	$q_{\mathrm{ad},e}=K_{F}\cdot \gamma_{\mathrm{ads},e}^{\frac{1}{n}}$	$\log q_{\mathrm{ad},e}=\frac{1}{n}\log\gamma_{\mathrm{ads},e}\;+\;\log K_{F}$	$\log q_{\mathrm{ad},e}=f\left(\log\gamma_{\mathrm{ads},e}\right)$
Dubinin–Radushkevich	$q_{\mathrm{ad},e}=q_{s}\cdot \exp\left(\cdot K_{\mathrm{DR}}\cdot \varepsilon_{\mathrm{DR}}^{2}\right)$, $\varepsilon_{DR}=R\cdot T\cdot \ln\left(1+\frac{1}{\gamma_{\mathrm{ad}s,e}}\right)$	$\ln q_{\mathrm{ad},e}=\ln q_{s}-K_{\mathrm{DR}}\cdot \varepsilon_{\mathrm{DR}}^{2}$	$\ln q_{\mathrm{ad},e}=f\left(\varepsilon_{\mathrm{DR}}^{2}\right)$
Temkin	$q_{\mathrm{ad},e}=\frac{R\cdot T}{b_{T}}\cdot \ln\left(A_{T}\gamma_{\mathrm{ad}s,e}\right)$	$q_{\mathrm{ad},e}=\frac{RT}{b_{T}}\ln\gamma_{\mathrm{ad}s,e}\;+\;\frac{RT}{b_{T}}\ln A_{T}$	$q_{\mathrm{ad},e}=f\left(\ln\gamma_{\mathrm{ads},e}\right)$
